# Identifying Barriers to Effective Cancer Pain Management in Oman: Implications for Palliative Care

**DOI:** 10.3390/curroncol31060225

**Published:** 2024-05-24

**Authors:** Husain Ali Alaswami, Atika Ahmed Al Musalami, Muaeen Hamed Al Saadi, Adhari Abdullah AlZaabi

**Affiliations:** 1 College of Medicine and Health Sciences, Sultan Qaboos University, Muscat 123, Omans130684@student.squ.edu.om (M.H.A.S.); 2 National Cancer Center, The Royal Hospital, Muscat 131, Oman; 3 Human and Clinical Anatomy Department, College of Medicine and Health Sciences, Sultan Qaboos University, Muscat 123, Oman

**Keywords:** analgesics, pain management, perception, attitude to health, palliative care

## Abstract

Background: Effective cancer pain management is essential for improving the quality of life of patients. However, the use of analgesics is often suboptimal due to various patient-related barriers. This study aims to explore the perceptions, knowledge, and attitudes toward analgesic use among cancer patients in Oman, which may influence their pain management strategies. Methods: In a cross-sectional study, we assessed 68 cancer patients undergoing pain management at an inpatient cancer clinic of a tertiary hospital in Oman from a pool of 154 eligible participants. The Barriers Questionnaire (BQ) and the Patient Pain Questionnaire (PPQ), both Arabic versions, were administered to evaluate the patients’ barriers to cancer pain management. The study period and the criteria for patient selection are specified. Results: With a participation rate of 44.2% and a female-to-male ratio of 2.28:1, the mean score on the BQ was 2.52 (SD 0.84), indicating a moderate level of perceived barriers. Patients’ scores suggested notable barriers, with older patients exhibiting reluctance toward analgesics for fear of masking symptoms and female patients expressing greater concerns about developing drug tolerance. Conclusion: The findings highlight significant attitudinal barriers to effective cancer pain management in Oman, notably a prevalent fear of medication tolerance. The study stresses on the need for targeted patient education and the correction of misconceptions. It also points to the influence of cultural and religious beliefs on patient responses, advocating for the implementation of culturally sensitive, evidence-based pain management guidelines, and the support of multidisciplinary palliative care teams.

## 1. Introduction

Despite the advancements in cancer pain management (CPM) and the guidelines issued by the World Health Organization (WHO), pain continues to be one of the most frequently reported symptoms during and after cancer treatment [1,2]. Almost 55.0% of patients on anticancer therapy suffer from pain, and 39.3% of patients reported pain after completing treatment [1]. The majority of these patients are in the later stages of the disease and one-third of them reported experiencing moderate to severe pain. Previous reports from Oman indicated that approximately 36% of admissions to oncology wards are primarily for pain management [3]. Pain intensity, frequency, and distress are highly associated with lower overall quality of life (QoL) [4]. In addition, uncontrolled cancer pain was correlated with major functional disability, thus leading to interference with patients’ daily activities [4,5]. Moreover, the impact of cancer pain on quality of life can extend beyond the patients to include their families and caregivers [6]. Therefore, the issue of managing cancer-related pain should not be underestimated.

The issue of inappropriate CPM is complex as it depends on multiple factors related to the patients, caregivers, and health workers. In fact, prior studies reported that patients’ pain usually is inadequately controlled due to multiple patient-related factors such as patients’ pain knowledge, sociodemographic characteristics, fear of addiction, and side effects of analgesics [7]. Attitudinal barriers to pain management are important as they are often based on misconceptions about pain and pain management. Patients who experience pain might choose not to use the resources available to manage pain because of erroneous beliefs. The Barriers Questionnaire (BQ) is the gold standard tool to measure patients’ attitudes toward CPM [8]. Studies have revealed several barriers that hinder the proper use of CPM among patients such as the fear of building tolerance, the fear of addiction, and the fear of masking a new pain [9]. Some patients think that physicians should focus more on curing the disease rather than trying to mitigate the pain [9]. Fear of addiction was the main reason to refuse strong opioids among 50% of Turkish cancer patients. Thirty-six percent of cancer patients in Turkey preferred non-opioid drugs for CPM [10]. Pain management and patients’ attitudes are linked with sociocultural beliefs. Therefore, understanding and resolving these barriers in different contexts would help improve patients’ attitudes, enhance willingness to report pain accurately and take analgesics, and improve patients’ quality of life [11].

In Oman, there is a great move toward the establishment of palliative care services due to the increased number of patients diagnosed at advanced stages. Palliative care services aim mainly to improve the quality of care of patients and manage their pain and other needs properly. Therefore, evaluating the attitudinal barriers to proper cancer management among patients and healthcare providers is critical in order to plan a proper intervention to improve the intervention outcome. Evaluation of nurses’ knowledge and attitude toward cancer pain management in Oman revealed gaps in knowledge that necessitate proper intervention. No prior study from Oman has investigated the attitudinal barriers and misconceptions among cancer patients regarding pain management. Thus, this study aims to investigate the patients’ related barriers to cancer pain management in Oman. 

## 2. Methods and Study Design

### 2.1. Setting and Participants

This is a cross-sectional survey-based study conducted at the National Oncology Center in the Royal Hospital, which is a tertiary teaching hospital in Muscat, Oman. Cancer patients were included in the study if they were Omani, 18 years or older, aware of their cancer diagnosis, had experienced cancer-related pain, were or currently taking oral analgesics, and could read and write. Cancer patients who were admitted to the intensive care unit (ICU) or those on pain management after a surgical procedure were excluded. Due to the restricted inclusion criteria for our study—specifically targeting cancer patients who were both aware of their diagnosis and currently undergoing pain management—the feasibility of achieving a calculated sample size based on general population parameters was challenging.

The dynamic nature of patient admissions and discharges resulted in the sample size varying day by day. To address this, we collaborated closely with the nursing staff and treating physicians to identify all eligible patients admitted during the study period. This approach ensured a comprehensive inclusion of the potential study population under the stringent criteria set forth for participation.

Despite these constraints, we were able to achieve a response rate of 44%, which we believe is substantial given the context of our study’s specific patient population. It is also noteworthy that our respondents represent a diverse and relevant cross-section of the patient population meeting the study’s inclusion criteria, which contributes valuable insights to the subject matter.

Ethical approval was obtained from the research ethics committee at the Ministry of Health (SRC#60/2021).

### 2.2. Study Instruments

A self-administered questionnaire was disseminated to all consented patients. The questionnaire included the following sections:

#### 2.2.1. Demographic and Disease-Related Information Questionnaires

The patients’ sociodemographic data include gender, age, education, marital status, career, and their family’s average monthly income. In addition, the disease-related information consists of the disease diagnosis, metastasis status, surgery, chemotherapy, and radiotherapy.

#### 2.2.2. Barriers Questionnaire II-12 (BQII-12) (12 Items)

The original Barrier Questionnaire (BQ) was developed by Ward et al. in 1994 [12]. It is a 27-item self-reported instrument used to test 8 concerns that are thought to be barriers to an effective CPM. The barriers are addiction, tolerance, side effects, fatalism, being good, distracting the healthcare worker, disease progression, and fear of injections. The internal consistency of this questionnaire is 0.89. The BQII is the most used questionnaire to address barriers to effective CPM and is considered the gold standard for this purpose [13]. In 2018, a shortened version of the BQII was developed by Koller et al. to eliminate the redundancy of some questions and decrease the burden on the patients [14]. The BQII-12 is the new version with a reduced number of items from 27 to 12 while maintaining a high internal consistency. These items are rated on a 6-point Likert-type scale, from 0 (do not agree at all) to 5 (agree very much), with higher scores indicating stronger barriers. Mean scores for the total BQII-12 are commonly used in analyses.

#### 2.2.3. Patient Pain Questionnaire (PPQ) (16 Items)

The PPQ is a 16 items self-report questionnaire designed by the City of Hope to test the knowledge and experience of a cancer patient [15]. The patients answer a Likert-type scale questionnaire on two major themes: the knowledge of the patient about cancer pain and pain relief (9 items) and their personal experience with pain (7 items). All items are designed such that picking 0 is the most positive outcome and 10 is the most negative one. The PPQ was tested by the original authors for validity and to establish reliability. The content validity of the PPQ is 95%, and the internal consistency is 74%.

All questions in both questionnaires have been translated and back-translated into Arabic. A pilot study was conducted on 15 cancer patients who revealed a good inter-item reliability of the translated survey (Cronbach alpha of all sections >0.7). 

#### 2.2.4. Statistics

All data were collected using a Microsoft Excel 2018 (Redmond, WA, USA) spreadsheet. The analyses were conducted using IBM Statistical Package for Social Sciences (SPSS) software (Version 27.0; IBM Corp., New York, NY, USA). Statistical descriptions of the demographic and disease-related information, pain knowledge, and attitudes toward pain management were generated using case numbers, constituent ratios, and rates. The one-way analysis of variance (ANOVA) has been used to compare the differences in the attitude scores of patients among several characteristics. The significance of the association between different factors and mass size has been measured using the Chi-square test to test the importance of the association between classified variables. *p* < 0.05 is considered statistically significant.

## 3. Results

### 3.1. Descriptive Statistics

A total of 68 patients participated in the study. The majority of the patients were female (69%), with a female-to-male ratio of 2.3:1. The mean age was 43.2 years (SD = 11.6), ranging from 18 to 74 years. The majority of patients (54%) completed high school (12 years of education) as their highest level of education, followed by patients who can only read and write (19%), had a university degree (11%), received primary education (10%), and higher degree (3%). Most patients were married (72%). The majority of patients interviewed were breast cancer patients (36.8%), followed by gastrointestinal tumor-related tumors (26.5%) and uro-reproductive cancers (16.2%). More than half of the patients (64.7%) had metastasis at the time of diagnosis (64.7%). In addition, most patients (67.4%) underwent surgical procedures in the past. Other sociodemographic data and the disease characteristics of the patients are presented in Table 1.

### 3.2. Response to Barriers Questionnaire

**Figure 1:**
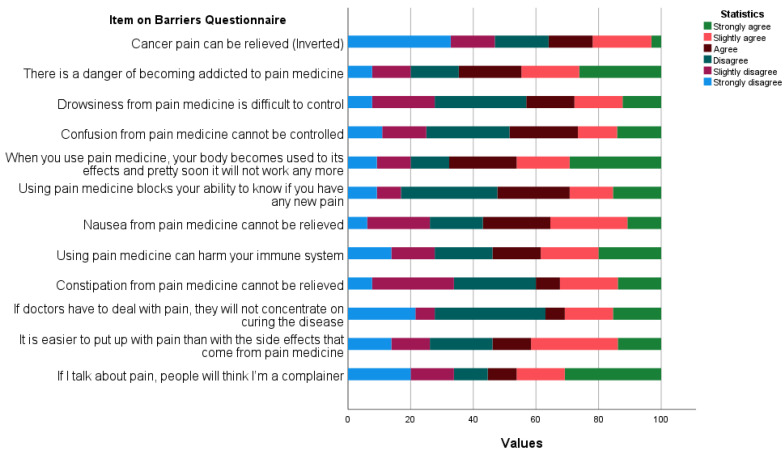
Distribution of responses among each Barriers Questionnaire item.

The distribution of responses among each Barriers Questionnaire item is shown in Figure 1. In all items aside from item one “Cancer pain can be relieved”, agreeing with the statements implies poor outcomes. A dominant majority of patients (64%) believe that cancer pain can be relieved; however, slightly more than half of patients (55%) agreed that talking about the pain will make others think they are complainers. The most agreed-upon items are those relating to the possibility of getting addicted to pain medications (64%) and developing a tolerance for analgesics (67%).

Table 1 and Table 2 show the total score for the domains and subscales, respectively. As the total BQ score ranges from 0 to 5, a score of 2.5 or higher is considered high and indicates a high level of perceived barriers to effective pain management. The mean patients’ score on the BQ was 2.52 (SD = 0.84, CI 2.31–2.73) which indicates that patients hold a considerable number of barriers hindering effective pain management (score > 2.5).

The three domains with the highest mean scores were tolerance (3.17 ± 1.68), addiction (3.06 ± 1.65), and the desire to be good (2.75 ± 1.92). The highest subscale was for fear of harmful effects (2.84 ± 1.37), with high concerns regarding tolerance and addiction. The distraction and fatalism subscales were not well endorsed, with a mean of 1.95 ± 1.74 and 1.73 ± 1.57, respectively. Interestingly, all items on the questionnaire had a mean score > 1.5, which means a moderate to high level of concern regarding all barriers. Female patients scored higher overall on the Barriers Questionnaire (2.57 ± 0.88) when compared to their male counterparts (2.41 ± 0.73); however, no significant association was found (*p* > 0.05).

### 3.3. Relationship between the BQ Score and Other Variables

Potential correlations between the BQ components and other demographic variables were investigated using the ANOVA and independent *t*-test. While there were no notable associations between any of the demographic variables and the overall BQ score, three components of the BQ were significantly linked with three distinct demographic variables. A one-way ANOVA was performed to compare the effect of age groups on different variables on the BQ. A one-way ANOVA revealed that there was a statistically significant difference in the fatalism variable between at least two groups (F(2, 59) = 3.169, *p* = 0.049) as displayed in Table 3. Tukey’s HSD test shown in Table 4 found that the mean value of fatalism was significantly different between age group ≤44 (1.471 ± 1.561) and age group ≥ 60 (3.500 ± 2.380) with a *p* value = 0.040. There was no statistically significant difference between age groups and any other variables on the BQ.

The second correlation suggested that the female gender was associated with significantly worse perceived outcomes to developing tolerance to pain medications compared to the male gender, with scores 3.51 ± 1.51 and 2.40 ± 1.81, respectively (*t*(63) = 2.562, *p* = 0.013), with a difference of 1.111 (95% CI, 0.244 to 1.977) displayed in Table 5. The final correlation implied that cancer patients with metastasis were more likely to refuse analgesics for the fear of getting addicted to them, as indicated by their significantly higher scores of 3.41 ± 1.43 compared to their counterparts who scored 2.36 ± 1.84, respectively (*t*(34.434) = 2.349, *p* = 0.025), with a difference of 1.054 (95% CI, 0.143 to 1.967) (Table 6).

### 3.4. Response to the Patient Pain Questionnaire

The questionnaire uses a scale where selecting zero represents the most positive response, and selecting ten represents the most negative response. The patients’ score (mean ± SD) for the knowledge subscale was 5.21 ± 1.75, indicating that the patient population had poor pain knowledge. The items in the knowledge subscale that received the poorest responses were those related to the fear of developing tolerance: “It is important to give the lowest amount of medicine possible to save larger doses for later when the pain is worse” and “Pain medicines should be given only when pain is severe”, with scores (mean ± SD) of 6.10 ± 3.50 and 6.82 ± 3.34, respectively. These findings are consistent with the responses on the BQ, where tolerance was identified as the most significant barrier to pain management.

The score (mean ± SD) on the experience subscale for patients is slightly better at 4.97 ± 1.60. Patients reported a low level of current pain and a moderate level of pain experienced in the past week with scores of 2.08 ± 2.22 and 4.17 ± 3.20, respectively. A significant number of patients responded with “extremely” when asked whether their pain was distressing to them or their families, with scores of 5.93 ± 3.28 and 7.30 ± 3.36, respectively. The majority of patients (65.4%) reported feeling that they were not in control of their pain, with a score of 6.52 ± 2.84. However, a considerable proportion of patients (61.5%) believe that their pain will improve in the future.

## 4. Discussion

The increasing incidence of cancer within the Gulf Cooperation Council (GCC) region, particularly among individuals under 50, presents a significant challenge in Oman, as a majority of these cases are diagnosed at advanced stages [16]. These late diagnoses often result in aggressive treatment regimens with poor prognoses and complex, multifaceted complications. In response, Oman is committed to enhancing the quality of life for cancer patients by providing cutting-edge treatments and adopting the latest management guidelines across the entire cancer care continuum. Despite these efforts, the demand for comprehensive palliative care is growing due to the nuanced needs of patients in the advanced stages of their illness.

This cross-sectional study is the first to assess patient-related barriers to effective pain management in Oman contributing to a growing body of knowledge in a region where such data are scarce. This research highlights the persistence of attitudinal barriers, which pose a significant obstacle to effective cancer pain management in Oman, as reflected in the mean Barriers Questionnaire (BQ) score.

The observed barriers to pain management in our study resonate with patterns identified in the broader Middle Eastern context, suggesting a regional trend shaped by cultural and educational factors that influence patient attitudes. Our findings align with barrier levels documented in Jordan [17], Saudi Arabia [18], and the United Arab Emirates [19], highlighting a regional consistency potentially rooted in shared cultural and health beliefs. Notably, the Barriers Questionnaire (BQ) scores from this study exceed those from diverse cultural settings such as the United States, Turkey, and Ireland, suggesting that culture influences the perception and management of pain across different societies [20,21,22,23].

Interestingly, our findings also indicate that the setting plays a crucial role in the perception of barriers. In studies conducted in Western contexts such as the hospice setting in the Midwest and East Coast of the U.S. [22,23], the lower BQ scores suggest that a supportive environment, likely coupled with comprehensive patient education, can significantly reduce attitudinal barriers. This emphasizes the potential impact of tailored patient education and the need for a supportive healthcare environment in mitigating barriers to pain management.

Our findings resonate with prior findings that suggest that patient and caregiver beliefs distinctly influence responses to cancer management, a dynamic more evident upon examining the varied BQ subscales across studies [20,24,25,26]. The prevalent fear of medication tolerance necessitates targeted interventions that address and correct misconceptions about pain medication use. A comprehensive approach involving healthcare providers, educators, and policymakers is crucial to shift patient and caregiver beliefs and improve outcomes. Efforts should be made to implement evidence-based guidelines that respect cultural sensibilities while ensuring that patients receive the best possible care. 

The relatively high scores on the harmful effects subscale reveal attitudinal patterns that undermine efforts toward efficient cancer pain relief.

Hesitancy to use analgesics stemming from fear of tolerance, addiction, or fear of opioid crisis must be combated, as with all barriers, by robust patient education and the correction of false understandings held by patients and their caregivers [25,27]. The influence of beliefs the caregivers hold has been shown to significantly impact the patients’ attitudes toward the different barriers and the feeling of being stigmatized by the regular use of opioids. 

In this study, the lowest-ranked barrier was fatalism, which implies that patients do not strongly believe that pain is an inherent part of cancer and cannot be relieved. These findings align with Islamic perspectives, which prioritize the mitigation of suffering and endorse proactive treatment to relieve pain and illness [28]. The minimal endorsement of fatalism here is consistent with the tenets of Islamic jurisprudence, which not only permit but often necessitate the pursuit of therapeutic interventions to ease discomfort and disease.

The subscale related to physiological effects also reported a low score primarily attributed to concerns about potential immunological reactions to pain medications and the masking of consequential pain. Despite several studies demonstrating that the chance of addiction and negative physiological effects is minimal, many patients still decline to take sufficient pain relievers due to this reason. This hesitancy has been previously documented, where patients favored non-opioid over potent opioid analgesics, driven by fears of addiction, religious convictions, and cultural prohibitions [11]. Moreover, some patients claimed they would rather experience pain than deal with side effects such as nausea, vomiting, constipation, or drowsiness [29].

The moderate score in the communication subscale suggests a tendency among patients to be perceived as good patients who do not burden healthcare providers with their pain, potentially diverting attention from the primary disease. This identifies an area for intervention where healthcare providers must foster open lines of communication, encouraging patients to report pain without fear of being burdensome.

In conclusion, this study advocates for a paradigm shift in cancer pain management in Oman, recognizing the critical role of patient education, culturally sensitive communication, and policy reforms in overcoming attitudinal barriers. It calls for an integrated effort to reshape perceptions of pain management, ultimately enhancing the quality of life for cancer patients.

## 5. Limitations

This study is unique in that there have been no similar studies conducted in Oman, so it serves as a valuable baseline for future studies. However, one major limitation of the study is the convenient sample used, which may introduce selection bias and affect the study’s external validity. Additionally, the study was conducted in a single center, specifically an inpatient cancer ward at a large tertiary governmental hospital that emphasizes proper pain education and analgesic use. Furthermore, the inherent nature of cross-sectional studies restricts the ability to infer causality. Therefore, it is important to investigate other settings to determine if barriers differ between them. While the overall Barriers Questionnaire (BQ) demonstrated acceptable internal consistency, certain domains such as “Tolerance” and “Fatalism” exhibited significantly low Cronbach’s alpha values, indicating potential issues with internal consistency within these subscales. This suggests that the items in these domains may not be coherently measuring the constructs they are intended to assess. 

## 6. Conclusions

The study reveals that there are strong and widespread attitudinal barriers to effective cancer pain management in Oman, with the fear of developing tolerance to pain medications being the most prevalent barrier. The harmful effects subscale also demonstrated poor attitudinal behaviors that nullify efforts to achieve effective cancer pain suppression. Fatalism was the lowest-ranked barrier in this study, implying that patients do not strongly believe that pain is an inherent part of cancer and cannot be relieved. The study’s results suggest that proper patient education and the correction of false understandings by patients or their caregivers are necessary to combat these barriers. Furthermore, the study highlights the importance of cultural and religious context in influencing patients’ responses toward their disease and, by extension, their responses to treatment. Finally, the study recommends the implementation of evidence-based guidelines and policies for pain management, the need for multidisciplinary teams, and the importance of palliative care for cancer patients in Oman.

## Figures and Tables

**Table 1:** Sociodemographic and disease characteristics of the patients (*n* = 68) (BQ: Barriers Questionnaire).

Item	*n*	Proportion (%)	Attitude Score on BQ	
			Mean	SD
**Gender**				
Men	21	30.88	2.41	0.73
Women	47	69.11	2.57	0.88
**Age (Years)**				
≤44	36	53.73	2.40	0.87
45–59	27	40.30	2.68	0.82
≥60	4	5.97	2.52	0.61
**Education level**				
Read and write	13	19.11	2.42	0.61
Primary education	7	10.29	2.30	1.05
High school diploma	37	54.41	2.54	0.91
Bachelor’s degree	8	11.76	2.77	0.82
Higher education	2	2.94	2.58	0.94
Missing	1	1.47	-	-
**Marital Status**				
Single	12	17.65	2.92	0.62
Married	49	72.05	2.44	0.87
Divorced	5	7.35	2.83	0.29
**Primary Tumor**				
Breast cancer	24	36.76	2.34	1.00
Oral, nasopharyngeal, esophageal, and gastrointestinal cancers	18	26.47	2.66	0.49
Kidney, ureter, bladder, ovarian cervical and uterine cancers	10	16.17	2.53	1.00
Lung cancer	2	2.94	2.67	1.30
Liver, pancreatic, and lymphoma	1	1.47	3.17	-
Other	11	16.17	2.62	0.67
**Metastasis**				
No	24	35.29	2.23	0.89
Yes	44	64.71	2.64	0.78
**Previous Surgeries**				
No	24	35.29	2.60	0.84
Yes	44	64.70	2.47	0.84

**Table 2:** Average scores for each domain of attitude toward cancer pain management for the patients.

Domains	Patient		
	Score (Mean ± SD)	Rank	Cronbach’s α *
Tolerance	3.17 ± 1.68	1	0.30
Addiction	3.06 ± 1.65	2	0.45
Be Good	2.75 ± 1.92	3	0.46
Immune	2.63 ± 1.73	4	0.67
Masking	2.57 ± 1.68	5	0.60
Symptoms	2.48 ± 0.87	6	0.67
Distraction	1.95 ± 1.74	7	0.36
Fatalism	1.73 ± 1.57	8	0.04
BQ Total	2.52 ± 0.84	-	0.73
Male	2.41 ± 0.73		
Female	2.57 ± 0.88		

Footnotes: *n* = 63. Response rate: 92.6% * Cronbach’s α > 0.700 is considered significant.

**Table 3:** ANOVA analysis between age categories and variables of the Barriers Questionnaire.

Variable		Sum of Squares	df	Mean Square	F	Sig.
BQTotal	Between Groups	1.517	2	0.759	1.100	0.340
	Within Groups	40.687	59	0.690		
	Total	42.204	61			
Symptoms	Between Groups	3.411	2	1.705	2.308	0.108
	Within Groups	44.326	60	0.739		
	Total	47.737	62			
Tolerance	Between Groups	13.850	2	6.925	2.599	0.083
	Within Groups	162.509	61	2.664		
	Total	176.359	63			
Masking	Between Groups	11.763	2	5.882	2.140	0.126
	Within Groups	167.674	61	2.749		
	Total	179.437	63			
Fatalism	Between Groups	14.942	2	7.471	3.169	* 0.049
	Within Groups	141.471	60	2.358		
	Total	156.413	62			
Distraction	Between Groups	2.340	2	1.170	0.373	0.691
	Within Groups	191.597	61	3.141		
	Total	193.938	63			
BeGood	Between Groups	0.013	2	0.007	0.002	0.998
	Within Groups	232.924	61	3.818		
	Total	232.937	63			
Addiction	Between Groups	2.592	2	1.296	0.465	0.630
	Within Groups	170.017	61	2.787		
	Total	172.609	63			

* Significant at 0.05 level.

**Table 4:** Results of the post hoc test.

Dependent Variable	(I) Age Categorized	(J) Age Categorized	Mean Difference (I-J)	Std. Error	Sig.	95% Confidence Interval	
						Lower Bound	Upper Bound
Fatalism	≤44	45–59	−0.330	0.405	0.696	−1.30	0.642
		≥60	−2.030 *	0.811	* 0.040	−3.98	−0.078
	45–59	≤44	0.330	0.404	0.696	−0.642	1.301
		≥60	−1.700	0.826	0.108	−3.687	0.287
	≥60	≤44	2.030 *	0.811	* 0.040	0.0788	3.980
		45–59	1.700	0.826	0.108	−0.287	3.687

* The mean difference is significant at the 0.05 level.

**Table 5:** Independent samples *t*-test results of tolerance compared between genders.

Dependent Variable		Levene’s Test for Equality of Variance		*t*-Test for Equality of Means						
		F	Sig.	*t*	df	(Sig.)	Mean Difference	Std. Error Difference	95% Confidence Interval of the Difference	
									Lower	Upper
Tolerance	Equal variances assumed	2.309	0.134	2.562	63	0.013	1.111	0.434	0.244	0.244
	Equal variances not assumed			2.390	31.283	0.023	1.111	0.465	0.163	2.059

**Table 6:** Independent samples *t*-test results of addiction compared between metastasis status.

Dependent Variable		Levene’s Test for Equality of Variance		*t*-Test for Equality of Means						
		F	Sig.	*t*	df	Sig.	Mean Difference	Std. Error Difference	95% Confidence Interval of the Difference	
									Lower	Upper
Addiction	Equal variances assumed	5.751	0.019	2.545	63	0.013	1.054	0.415	0.226	1.883
	Equal variances not assumed			2.349	34.434	0.025	1.054	0.450	0.143	1.967

## Data Availability

Data are available upon request.

## References

[1] Van Den Beuken-Van Everdingen M.H.J., Hochstenbach L.M.J., Joosten E.A.J., Tjan-Heijnen V.C.G., Janssen D.J.A. (2016). Update on Prevalence of Pain in Patients with Cancer: Systematic Review and Meta-Analysis. J. Pain Symptom Manag..

[2] Nardelli C., Granata I., Nunziato M., Setaro M., Carbone F., Zulli C., Pilone V., Capoluongo E.D., De Palma G.D., Corcione F. (2021). 16S rRNA of Mucosal Colon Microbiome and CCL2 Circulating Levels Are Potential Biomarkers in Colorectal Cancer. Int. J. Mol. Sci..

[3] Faris M., Al-Bahrani B., Emam Khalifa A., Ahmad N. (2007). Evaluation of the prevalence, pattern and management of cancer pain in Oncology Department, The Royal Hospital, Oman. Gulf J. Oncolog..

[4] Rodriguez C., Ji M., Wang H.L., Padhya T., Mcmillan S.C. (2019). Cancer Pain and Quality of Life. J. Hosp. Palliat. Nurs..

[5] te Boveldt N., Vernooij-Dassen M., Burger N., Ijsseldijk M., Vissers K., Engels Y. (2013). Pain and its interference with daily activities in medical oncology outpatients. Pain Physician.

[6] Raphael J., Hester J., Ahmedzai S., Barrie J., Farqhuar-Smith P., Williams J., Urch C., Bennett M.I., Robb K., Simpson B. (2010). Cancer Pain: Part 2: Physical, Interventional and Complimentary Therapies; Management in the Community; Acute, Treatment-Related and Complex Cancer Pain: A Perspective from the British Pain Society Endorsed by the UK Association of Palliative Medicine and. Pain Med..

[7] Lin C.C., Chou P.L., Wu S.L., Chang Y.C., Lai Y. (2006). Long-term effectiveness of a patient and family pain education program on overcoming barriers to management of cancer pain. Pain.

[8] Jacobsen R., Møldrup C., Christrup L., Sjøgren P. (2009). Patient-related barriers to cancer pain management: A systematic exploratory review. Scand. J. Caring Sci..

[9] Al Qadire M. (2012). Patient-related barriers to cancer pain management in Jordan. J. Pediatr. Hematol. Oncol..

[10] Colak D., Oguz A., Yazilitas D., Imamoglu I.G., Altinbas M. (2014). Morphine: Patient knowledge and attitudes in the central Anatolia part of Turkey. Asian Pacific J. Cancer Prev..

[11] Makhlouf S.M., Pini S., Ahmed S., Bennett M.I. (2020). Managing Pain in People with Cancer—A Systematic Review of the Attitudes and Knowledge of Professionals, Patients, Caregivers and Public. J. Cancer Educ..

[12] Ward S.E., Hernandez L. (1994). Patient-related barriers to management of cancer pain in Puerto Rico. Pain.

[13] Jacobsen R., Liubarskiene Z., Møldrup C., Christrup L., Sjøgren P., Samsanavièiene J. (2009). Barriers to cancer pain management: A review of empirical research. Medicina.

[14] Koller A., Jahn P. (2018). Developing a Short Form of the German Barriers Questionnaire II: A Validation Study in Four Steps. J. Pain Symptom Manag..

[15] (2012). City of Hope The Patient Pain Questionnaire (PPQ). https://www.cityofhope.org/sites/www/files/2022-05/patient-pain-management.pdf.

[16] Al Zaabi A., Al Shehhi A., Sayed S., Al Adawi H., Al Faris F., Al Alyani O., Al Asmi M., Al-Mirza A., Panchatcharam S., Al-Shaibi M. (2023). Early Onset Colorectal Cancer in Arabs, Are We Dealing with a Distinct Disease?. Cancers.

[17] Al-Atiyyat N.M.H., Vallerand A.H. (2018). Patient-related attitudinal barriers to cancer pain management among adult Jordanian patients. Eur. J. Oncol. Nurs..

[18] Alodhayani A., Almutairi K.M., Vinluan J.M., Alsadhan N., Almigbal T.H., Alonazi W.B., Batais M.A. (2021). Gender Difference in Pain Management Among Adult Cancer Patients in Saudi Arabia: A Cross-Sectional Assessment. Front. Psychol..

[19] Obaid A., Al Hroub A., Al Rifai A., Alruzzieh M., Radaideh M., Tantawi Y. (2023). Barriers to Effective Cancer Pain Management, Comparing the Perspectives of Physicians, Nurses, and Patients. Pain Manag. Nurs..

[20] Lou F., Shang S. (2017). Attitudes towards pain management in hospitalized cancer patients and their influencing factors. Chinese J. Cancer Res..

[21] Baǧçivan G., Tosun N., Kömürcü Ş., Akbayrak N., Özet A. (2009). Analysis of Patient-Related Barriers in Cancer Pain Management in Turkish Patients. J. Pain Symptom Manag..

[22] Ward S.E., Goldberg N., Miller-McCauley V., Mueller C., Nolan A., Pawlik-Plank D., Robbins A., Stormoen D., Weissman D.E. (1993). Patient-related barriers to management of cancer pain. Pain.

[23] Ward S.E., Berry P.E., Misiewicz H. (1996). Concerns about Analgesics among Patients and Family Caregivers in a Hospice Setting. Res. Nurs. Health.

[24] Silbermann M., Hassan E.A. (2011). Cultural perspectives in cancer care: Impact of Islamic traditions and practices in Middle Eastern Countries. J. Pediatr. Hematol. Oncol..

[25] Mercadante S., Adile C., Tirelli W., Ferrera P., Penco I., Casuccio A. (2021). Barriers and Adherence to Pain Management in Advanced Cancer Patients. Pain Pract..

[26] Lee B.O., Liu Y., Wang Y.H., Hsu H.T., Chen C.L., Chou P.L., Hsu W.C. (2018). Mediating Effect of Family Caregivers’ Hesitancy to Use Analgesics on Homecare Cancer Patients’ Analgesic Adherence. J. Pain Symptom Manag..

[27] Meghani S.H., Wool J., Davis J., Yeager K.A., Mao J.J., Barg F.K. (2020). When Patients Take Charge of Opioids: Self-Management Concerns and Practices Among Cancer Outpatients in the Context of Opioid Crisis. J. Pain Symptom Manag..

[28] Choong K.A. (2015). Islam and palliative care. Glob. Bioeth..

[29] Torresan M.M., Garrino L., Borraccino A., Macchi G., De Luca A., Dimonte V. (2015). Adherence to treatment in patient with severe cancer pain: A qualitative enquiry through illness narratives. Eur. J. Oncol. Nurs..

